# Moxibustion for diarrhea-predominant irritable bowel syndrome: A protocol for systematic review and network meta-analysis

**DOI:** 10.1097/MD.0000000000028373

**Published:** 2021-12-23

**Authors:** Tiantian Dong, Xuhao Li, Xin Ma, Xiqing Xue, Yi Hou, Yuanxiang Liu, Jiguo Yang

**Affiliations:** a Center for External Treatment of Traditional Chinese Medicine, Affiliated Hospital of Shandong University of Traditional Chinese Medicine, Jinan, People's Republic of China; b College of Acupuncture and Massage, Shandong University of Traditional Chinese Medicine, Jinan, People's Republic of China; c College of First Clinical Medicine, Shandong University of Traditional Chinese Medicine, Jinan, People's Republic of China.

**Keywords:** diarrhea-predominant irritable bowel syndrome, moxibustion, network meta-analysis, protocol, systematic review, traditional Chinese medicine

## Abstract

**Background:**

Irritable bowel syndrome (IBS) is commonly accompanied by intestinal dysfunction, and diarrhea-predominant irritable bowel syndrome accounts for approximately 23.4% of all cases of IBS. The purpose of this study was to evaluate the efficacy and safety of moxibustion in the treatment of diarrhea-predominant irritable bowel syndrome.

**Methods:**

According to the retrieval strategies, randomized controlled trials (RCTs) on moxibustion therapies for IBS-D will be obtained from the China National Knowledge Infrastructure, WanFang Data, Chinese Scientific Journals Database, PubMed, Embase, and Cochrane Library, regardless of publication date or language. Studies will be screened based on inclusion and exclusion criteria, and the Cochrane risk bias assessment tool will be used to evaluate the quality of the literature. The network meta-analysis will be performed with the Markov chain Monte Carlo method and carried out with Stata 14.2 and WinBUGS 1.4.3 software. Ultimately, the quality of the evidence obtained from the results will be evaluated.

**Results:**

This study will evaluate whether moxibustion therapy can effectively treat diarrhea-predominant irritable bowel syndrome.

**Conclusion:**

This study will provide evidence for whether moxibustion therapy is beneficial to the treatment of human diarrhea-predominant irritable bowel syndrome.

**INPLASY registration number:**

INPLASY202180003.

## 1 Introduction

Irritable bowel syndrome (IBS) is commonly accompanied by intestinal dysfunction. IBS has a prevalence ranging from 1.1% to 29.2% in the whole population according to the Rome III criteria, with the diarrhea-predominant type accounting for approximately 23.4% of all cases.^[1]^ The disease is often treated with oral drugs, but the symptoms easily or intermittently after drug withdrawal, and it is difficult to cure, which affects the quality of life of patients.^[2]^ Many patients have refractory irritable bowel syndrome and are looking for complementary therapies that may be effective and less likely to have side effects.^[3]^

Patients with diarrhea-predominant irritable bowel syndrome (IBS-D) not only have to endure the pain of the disease but also bear more medical costs than patients without the disease.^[4,5]^ This finding indicates that the treatment of the disease is particularly important for improving the health and quality of life of patients. To date, abnormal intestinal motility, visceral hypersensitivity, abnormal neurohormonal response to stimulation or stress, and changes in normal intestinal flora are the causes of IBS-D.^[4]^ Recent conventional treatments, such as antispasmodics, fiber supplements, and antidepressants, have focused on alleviating (or alleviating) the symptoms of irritable bowel syndrome, but their effects are limited, which makes many patients with irritable bowel syndrome need complementary and alternative medicine. Acupuncture-related interventions are one of the most frequently sought complementary and alternative medicine modalities and have been widely used in various conditions, including functional gastrointestinal disorders, with 12 million treatments per year in the United States.^[6–9]^

Acupuncture includes acupuncture, moxibustion, and warm acupuncture. Moxibustion improves general health and treats chronic diseases such as arthritis and digestive system disorders by using the thermal stimulation produced by the burning of herbal preparations containing dried *Artemisia argyi* leaves or *A argyi* leaves on acupoints. Moxibustion is divided into direct moxibustion and indirect moxibustion.^[5]^ Among them, direct moxibustion involves placing the ignited moxa cone directly on the acupoint skin to ignite, which will cause pain and even scarring. Indirect moxibustion involves moxibustion of the ignited moxa cone at a certain distance from the skin, moxibustion of the cake made of salt, garlic, and traditional Chinese medicine, or hanging the moxa cone on the needle and igniting it for moxibustion.^[10]^

In recent years, there have been an increasing number of reports about moxibustion treatment of IBS-D, but there is no systematic review on moxibustion treatment of IBS-D. Therefore, we decided to fill the gap in the literature to provide experts and patients with up-to-date evidence that can be used to rigorously evaluate the effectiveness of this therapy and to guide clinical practice. We conducted this systematic review and meta-analysis to summarize the current evidence of the effects and safety of moxibustion therapy for the treatment of IBS-D.

## 2 Methods

### 2.1 Objectives and registration

This systematic review will aim to evaluate the effect and safety of moxibustion therapy for IBS-D. Our protocol has been registered on the International Platform of Registered Systematic Review and Meta-Analysis Protocols (INPLASY). The registration number was INPLASY202180003. All steps of this systematic review will be performed according to the Cochrane Handbook (5.2.0).

### 2.2 Ethics and dissemination plans

Given that there will be no patients recruited and no data gathered from patients, ethical approval is not necessary for our research. We will publish the results of this network meta-analysis in the form of journal papers or conference papers.

### 2.3 Eligibility criteria

Population, intervention, comparison, outcome and study design principles will be consulted to establish the inclusion and exclusion criteria of this systematic review.

#### 2.3.1 Types of participants

Participants who were diagnosed with diarrhea-predominant irritable bowel syndrome regardless of age, sex, and race. Diagnosis of IBS-D was based on specific diagnostic criteria (Rome I criteria, Rome II criteria, Rome III criteria, Rome IV criteria, or the Manning criteria).^[11]^

#### 2.3.2 Types of interventions and comparators

The series of moxibustion therapies involves many techniques, such as moxa stick moxibustion, moxa cone moxibustion, direct moxibustion, and indirect moxibustion. Moreover, many distinctive complex moxibustion manipulations are organically combined, such as partitioned moxibustion, moxa-moxibustion, and warm moxibustion.^[12]^ Studies that combine moxibustion with other therapies, such as acupuncture, massage, drugs, and physical interventions, will be included if they can prove that moxibustion is effective.

#### 2.3.3 Types of outcomes

The primary outcomes included the effective rate of clinical symptoms, IBS-D score, and total score on the gastrointestinal symptom rating scale (GSRS total score). The secondary outcomes will assess abdominal distension and the incidence of adverse events.

#### 2.3.4 Types of studies

The selected articles should be randomized controlled trials comparing moxibustion and control groups to evaluate the efficacy of moxibustion on IBS-D. We will include an assessment of moxibustion compared with control interventions, including inactive controls (such as placebo, no treatment) and active controls (such as drugs and acupuncture). Conference literature and papers, reviews, case series, case reports, experience summaries, and animal research will be excluded.

### 2.4 Data sources and retrieval strategy

We will search foreign and Chinese databases, including PubMed, EMBASE, MEDLINE, CENTRAL, CNKI, WanFang Data, CBM, and VIP from the inception of the coverage of these databases to July 2021.

Data, CBM, and VIP from the inception of the coverage of these databases to July 2020. The databases will be retrieved by combining the subject words with random words. Taking PubMed as an example, the retrieval strategy is shown in Table 1.

**Table 1:** Retrieval strategy of PubMed.

Number	Term
#1	“diarrhea-predominant irritable bowel syndrome” [mesh] or“Syndrome, Irritable Bowel, diarrhea-predominant” [title/abstract] or“Irritable Bowel Syndromes” [title/abstract] or“IBS-D” [title/abstract].
#2	“Moxibustion”[title/abstract] or“Moxibustion therapy”[title/abstract] or“herb partitioned moxibustion”[title/abstract] or“moxibustion with amugwort stick”[title/abstract] or“moxa cone moxibustion”[title/abstract]or“direct moxibustion ”[title/abstract]or“indirect moxibustion”[title/abstract].
#3	“randomized controlled trials”[mesh]) or“RCT”[title/abstract] or“controlled clinical trial”[mesh] or“randomized”[title/abstract] or“randomly”[title/abstract]“orrandom”[title/abstract] or“controlled”[title/abstract] or“control”[title/abstract] or “trial”[title/abstract].
#4	#1 and #2 and#3.

The search terms will be adapted appropriately to conform to the different syntax rules of the different databases.

### 2.5 Study selection and data extraction

**Figure 1:**
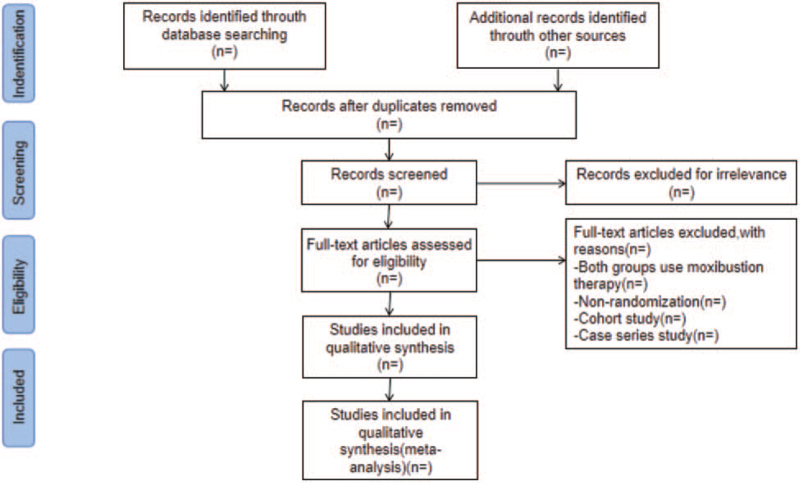
Preferred reporting items for systematic reviews and meta-analyses flow chart.

EndNote X9 (Toronto, ON, Canada) will be used to manage the retrieved studies. As shown in Fig. 1, the study selection will be divided into 2 steps and completed by 2 researchers (XL and XM). Preliminary screening: duplicate and irrelevant studies will be deleted while screening the titles and abstracts. Rescreening: we read through the full texts and select studies according to the inclusion and exclusion criteria.

According to the Cochrane Handbook for Systematic Reviews of Interventions, the 2 researchers (XX and YH) will extract data, including the author, publication time, participant number, age, race, lesion location, intervention measures, course of treatment and outcome indicators, and they will enter these data in the data extraction table to compare results.

### 2.6 Risk of bias assessment

Two researchers (XL and YH) will assess the quality of the included RCTs independently by utilizing the Cochrane Risk of Bias assessment tool. As specified by the Cochrane Handbook (5.2.0), the following sources of bias will be considered: random sequence generation, allocation concealment, participant blinding, outcome assessor blinding, incomplete outcome data, selective reporting, and other sources of bias. Each domain will be rated as having a high, low, or unclear risk of bias as appropriate.^[13]^ The 2 reviewers will resolve any disagreements through discussion, and a third reviewer (TD) will be consulted if no consensus is reached.

### 2.7 Statistical analysis

#### 2.7.1 Traditional meta-analysis

Direct comparisons of moxibustion efficacy will be performed using Review Manager 5.3. The outcomes will be mainly represented by the mean difference or odds ratio with 95% confidence intervals, and a *P* value <.05 will be considered significant. The Cochrane *Q* test and *I*^2^ statistics will be used to assess heterogeneity. When *P* < .1 or *I*^2^ > 50%, which indicates statistical heterogeneity, a random effects model will be used to calculate the outcomes; otherwise, a fixed effects model will be considered.

#### 2.7.2 Network meta-analysis

A network evidence diagram will be drawn to visually represent the comparisons between the studies. The size of the nodes represents the number of participants, and the thickness of the edges represents the number of comparisons. Stata 14.2 (Texas, USA) and WinBUGS 1.4.3 (Redmond, Washington) software will be used to carry out Bayesian network meta-analysis. Bayesian inference will be carried out using the Markov chain Monte Carlo method, the posterior probability will be inferred from the prior probability, and estimation and inference will be assumed when Markov chain Monte Carlo reaches a stable convergence state. As a result, the rank of the moxibustion effect will be presented by the surface under the cumulative ranking curve.

Inconsistencies between direct and indirect comparisons will be evaluated using the node splitting method.^[14]^ The choices between fixed effects and random effect models and between consistent and inconsistent models will be made by comparing the deviance information criteria for each model.^[15,16]^

#### 2.7.3 Subgroup and sensitivity analysis

If the heterogeneity is high, we will also perform subgroup analysis to calculate the combined statistics.^[17]^ The following subgroup analyses will be considered: sex, age, intervention time, intervention cycle, and course of the disease.

When sufficient data are available, sensitivity analysis will be performed to test the robustness of the primary outcomes, which includes assessing the quality of the methods, the quality of the studies, and the impact of sample size and missing data.

#### 2.7.4 Publication biases

If ≥10 studies are included, we will use funnel plots to assess the level of publication bias. Asymmetry in the funnel plot will suggest the possibility of small study effects, and the results of the analysis will be interpreted cautiously.

### 2.8 Quality of evidence

The Grading of Recommendations Assessment, Development and Evaluation (GRADE) system will be used to assess the overall quality of the evidence derived from the included studies.^[18]^ In addition, the results will be divided into high, moderate, low, and very low quality.

## 3 Discussion

Moxibustion, a very ancient modality of treating diseases, has been used throughout the history of human civilization and plays an important role in disease resistance. Moxibustion has been widely used for various conditions, including cancer, ulcerative colitis, stroke rehabilitation, constipation, hypertension, pain conditions, and breech presentation. Although moxibustion is frequently used for IBS-D in practice, there has been no systematic study to inform current evidence on the effectiveness of moxibustion treatment for IBS-D. We hope that the results of this study may provide evidence regarding the moxibustion treatment of IBS-D.

## Author contributions

**Conceptualization:** Tiantian Dong, Yuanxiang Liu, Jiguo Yang.

**Data curation:** Xuhao Li, Xin Ma, Xiqing Xue, Yi Hou.

**Formal analysis:** Tiantian Dong and Xuhao Li.

**Funding acquisition:** Jiguo Yang, Tiantian Dong.

**Investigation:** Jiguo Yang, Xin Ma.

**Methodology:** Yuanxiang Liu, Jiguo Yang, Yi Hou.

**Project administration:** Tiantian Dong.

**Software:** Xin Ma and Xiqing Xue.

**Supervision:** Yuanxiang Liu, Jiguo Yang, Xiqing Xue.

**Validation:** Jiguo Yang, Yuanxiang Liu.

**Visualization:** Jiguo Yang, Yuanxiang Liu.

**Writing – original draft:** Tiantian Dong.

**Writing – review & editing:** Yuanxiang Liu and Jiguo Yang.
